# Targeting tumour re-wiring by triple blockade of mTORC1, epidermal growth factor, and oestrogen receptor signalling pathways in endocrine-resistant breast cancer

**DOI:** 10.1186/s13058-018-0983-1

**Published:** 2018-06-08

**Authors:** Ricardo Ribas, Sunil Pancholi, Aradhana Rani, Eugene Schuster, Stephanie K. Guest, Joanna Nikitorowicz-Buniak, Nikiana Simigdala, Allan Thornhill, Francesca Avogadri-Connors, Richard E. Cutler, Alshad S. Lalani, Mitch Dowsett, Stephen R. Johnston, Lesley-Ann Martin

**Affiliations:** 1 The Breast Cancer Now Toby Robins Research Centre, The Institute of Cancer Research, London, SW7 3RP UK; 2 Centre for Cancer Imaging, The Institute of Cancer Research, Sutton, SM2 5NG UK; 3 Puma Biotechnology Inc., Los Angeles, CA USA; 4 The Ralph Lauren Centre for Breast Cancer Research, The Royal Marsden Hospital, London, SW3 6JJ UK; 5 Breast Unit, The Royal Marsden Hospital, London, SW3 6JJ UK

**Keywords:** Breast cancer, Oestrogen receptor, Neratinib, Everomilus, Endocrine resistance

## Abstract

**Background:**

Endocrine therapies are the mainstay of treatment for oestrogen receptor (ER)-positive (ER^+^) breast cancer (BC). However, resistance remains problematic largely due to enhanced cross-talk between ER and growth factor pathways, circumventing the need for steroid hormones. Previously, we reported the anti-proliferative effect of everolimus (RAD001-mTORC1 inhibitor) with endocrine therapy in resistance models; however, potential routes of escape from treatment via ERBB2/3 signalling were observed. We hypothesised that combined targeting of three cellular nodes (ER, ERBB, and mTORC1) may provide enhanced long-term clinical utility.

**Methods:**

A panel of ER^+^ BC cell lines adapted to long-term oestrogen deprivation (LTED) and expressing *ESR1*^*wt*^ or *ESR1*^*Y537S*^, modelling acquired resistance to an aromatase-inhibitor (AI), were treated in vitro with a combination of RAD001 and neratinib (pan-ERBB inhibitor) in the presence or absence of oestradiol (E2), tamoxifen (4-OHT), or fulvestrant (ICI182780). End points included proliferation, cell signalling, cell cycle, and effect on ER-mediated transactivation. An in-vivo model of AI resistance was treated with monotherapies and combinations to assess the efficacy in delaying tumour progression. RNA-seq analysis was performed to identify changes in global gene expression as a result of the indicated therapies.

**Results:**

Here, we show RAD001 and neratinib (pan-ERBB inhibitor) caused a concentration-dependent decrease in proliferation, irrespective of the *ESR1* mutation status. The combination of either agent with endocrine therapy further reduced proliferation but the maximum effect was observed with a triple combination of RAD001, neratinib, and endocrine therapy. In the absence of oestrogen, RAD001 caused a reduction in ER-mediated transcription in the majority of the cell lines, which associated with a decrease in recruitment of ER to an oestrogen-response element on the *TFF1* promoter. Contrastingly, neratinib increased both ER-mediated transactivation and ER recruitment, an effect reduced by the addition of RAD001. In-vivo analysis of an LTED model showed the triple combination of RAD001, neratinib, and fulvestrant was most effective at reducing tumour volume. Gene set enrichment analysis revealed that the addition of neratinib negated the epidermal growth factor (EGF)/EGF receptor feedback loops associated with RAD001.

**Conclusions:**

Our data support the combination of therapies targeting ERBB2/3 and mTORC1 signalling, together with fulvestrant, in patients who relapse on endocrine therapy and retain a functional ER.

**Electronic supplementary material:**

The online version of this article (10.1186/s13058-018-0983-1) contains supplementary material, which is available to authorized users.

## Background

Breast cancer (BC) is the most common malignancy in women, and was responsible for over 522,000 deaths in 2012 [1]. The majority of the BCs at primary diagnosis are oestrogen receptor (ER)-alpha positive (ER^+^) and depend on oestrogen (E) for their growth and progression. Endocrine therapies targeting oestrogenic stimulation of tumour growth have been developed clinically, and have shown success in reducing the mortality of ER^+^ BC. These therapies include: tamoxifen, which competes with E for the ER; fulvestrant (ICI182780), which binds to ER and targets it for degradation; and aromatase inhibitors (AIs), which block the conversion of androgens to E [2]. Despite the initial effectiveness of these approaches, many patients eventually relapse with either intrinsic or acquired resistance and, in most cases, continue to express ER [3, 4]. Studies suggest that *ESR1* mutations within the ligand binding domain of the receptor and/or cross-talk between ER and various cellular kinases allow the receptor to circumvent the need for steroid hormone [5]. In recent years, emphasis has been placed on co-targeting both the ER and the phosphatidylinositol-3-kinase/protein kinase B/mammalian target of rapamycin (PI3K/AKT/mTORC) pathway, known to phosphorylate and activate ER in a ligand-independent manner [6], to avoid or reverse these resistance mechanisms.

**Fig. 1:**
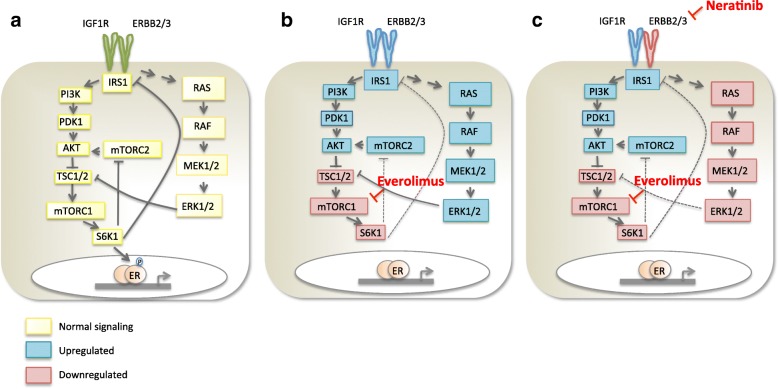
Simplified schematic diagram of the pathways described in this study. **a** Growth factor signalling (IGFR and ERBB) leads to activation of PI3K and phosphorylation of AKT. AKT inhibits TCS1/2, resulting in upregulation of mTORC1. In parallel, mTORC1 can also be upregulated by the RAS-RAF-MEK-ERK signalling pathway. ERK phosphorylates and inactivates TCS2 also leading to mTORC1 activation. S6 K1 activity increases as a result of mTORC1 activation. S6 K1 suppresses mTORC2 and IRS1. ER is also a target of S6 K1 leading to phosphorylation of serine 167. **b** Inhibition of mTORC1 with everolimus suppresses S6 K1 removing the negative feedback loop causing a rise in IRS1 and AKT activity via loss of suppression on mTORC2. Increased AKT activity suppresses TCS1/2 and increases expression of growth factor receptors (ERBB2/3) enhancing RAS-RAF-ERK signalling. **c** The dual blockade of ERBBs (neratinib) and mTORC1 signalling (everolimus) may suppress the two feedback loops described in **b**. Yellow shows normal mTORC signalling cascade; blue represents activated proteins; red represents inhibited proteins; dotted lines show loss of normal feedback loops

The combination of the rapalogue everolimus (RAD001) with exemestane, as third-line therapy in ER^+^/ERBB2-negative patients who relapsed on prior endocrine therapy, was reported from the BOLERO-2 trial to increase median progression-free survival (PFS) from 4.1 to 10.6 months compared with exemestane alone [7]. Nonetheless, it is clear that blockade of a single protein in a complex signalling cascade, even if a critical downstream effecter, is unlikely to provide a total or prolonged growth inhibition partly as a result of early rewiring. For instance, a negative feedback loop exists downstream in the PI3K/AKT/mTORC pathway such that mTORC1 inhibition leads to a reduction in S6 K1 activity, which in turn allows IRS1/2 expression to be increased with associated enhanced activation of IGFR1-dependent AKT activity [8]. Furthermore, mTORC1 blockade has also been shown to induce enhanced ERBB2/3 signalling [9], as well as ERK1/2 [8, 10], creating potential routes of escape negating the anti-tumour effectiveness of mTORC1 blockade and limiting long-term effectiveness (Fig. 1). This may account for the short-term clinical remissions and lack of stable disease, often with rebound growth at the time of further disease progression. As such, it is rational to explore targeting of mTORC1 with vertical blockade of growth factor receptors, such as those governing ERBB signalling (Fig. 1).

In this study, we assessed the effect of the combined therapy of the mTORC1 inhibitor, RAD001, with additional co-blockade of ERBB signalling with neratinib, an irreversible pan-ERBB receptor tyrosine kinase (RTK) inhibitor [11–13], in human BC cell models of endocrine-sensitive and -resistant disease with varying *ESR1*, *PIK3CA*, and *ERBB2* mutation status. Analysis showed that triple blockade of the three signalling nodes had greater efficacy than monotherapies both in vitro and in vivo and that the triple combination was well tolerated in a xenograft model.

## Methods

### Reagents

Primary antibodies against phospho-EGFR^tyr1068^ (CST-3777), total-EGFR (CST-2232), phospho-ERBB2^tyr1248^ (CST-2247), phospho-ERBB3^tyr1222^ (CST-4784), total-ERK1/2 (CST-9102), phospho-AKT^ser473^ (CST-9271), total-AKT (CST-9272), phospho-S6^ser240/244^ (CST-5364), total-S6 (CST-2217), phospho-ER^ser167^ (CST-5587), phospho-Rb^ser807/811^ (CST-8516), CDK4 (CST-2901), and cyclinD1 (CST-2922) were purchased from Cell Signaling, Inc.; total-ERBB3 (sc-415), ER-alpha (sc-8002, F-10), and PARP (sc-8007) were purchased from Santa Cruz; phospho-ERK1/2 and α-tubulin (T-9026) were obtained from Sigma; and total-ERBB2 was obtained from Millipore. Secondary antibodies (anti-mouse and anti-rabbit horseradish peroxidase) were obtained from Dako. 17β-Oestradiol (E2) and 4-hydroxytamoxifen (4-OHT) were purchased from Sigma, and fulvestrant (referred to as ICI) was obtained from Trocis, UK. Neratinib (PB272) was provided by Puma Biotechnology and Pfizer. Everolimus (RAD001) was purchased from Selleck. All chemicals, unless otherwise stated, were purchased from Sigma, UK. All tissue culture grade plastics were obtained from Nunc, UK.

### Tissue culture

The human BC cell lines were obtained from the ATCC Rockville, USA, or Asterand and authenticity was confirmed by STR. Cells were aliquoted to prevent phenotypic drift and routinely tested for *Mycoplasma* contamination. Wild-type (wt)-MCF7, wt-HCC1428, and wt-SUM44 cell lines were cultured in phenol red-free RPMI medium supplemented with 10% fetal bovine serum (FBS) and 1 nM E2. MCF7, HCC1428, and SUM44 cells adapted to long-term E deprivation (LTED) and modelling resistance to an AI were maintained in phenol red-free RPMI medium containing 10% dextran charcoal-stripped serum (DCC) in the absence of E2 [14]. Cells were passaged twice weekly and fed every 48 to 72 h. MCF7-LTED and HCC1428-LTED are homozygotes for *ESR1*^wt^, whilst SUM44-LTED are heterozygotes for *ESR1*^*Y537S*^.

### Cell proliferation assays

Wt-MCF7, wt-SUM44, wt-HCC1428, and their LTED derivatives were seeded in 10% DCC medium into 96-well plates. Cell monolayers were left to acclimatize for 24 h before treatment with the drug combinations for 6 days with a treatment change on day 3. Cell viability was determined using the CellTiter-Glo® Luminescent Cell Viability Assay (Promega) according to the manufacturer’s protocol.

### Transcription assays

Cell lines were seeded in 24-well plates in DCC medium and left to acclimatize for 24 h. The following day, transfection was performed using Fugene (Promega) with 0.1 μg of E response element linked luciferase (EREIItkluc) and 0.1 μg β-galactosidase (pCH110) reporter constructs [15]. Luciferase (Promega) and β-galactosidase (GalactoStar, Applied Biosystems) activity was measured using a luminometer.

### Western blotting

Whole cell extracts were generated as described previously [16]. Equal amounts of protein were resolved by SDS-PAGE and transferred to nitrocellulose membranes (Whatman). Antigen-antibody interactions were detected with Amersham ECL detection reagents (GE Healthcare).

### Chromatin immunoprecipitation

Wt-HCC1428 and HCC1428-LTED cells were cross-linked in 1% formaldehyde at room temperature for 10 min and then quenched with 125 mM glycine. Samples were then lysed and sonicated, and chromatin was immunoprecipitated by overnight incubation at 4 °C with ER (HC-20, sc-546) or IgG antibodies pre-bound with Protein G magnetic dynabeads (Invitrogen). Chromatin was washed vigorously with RIPA buffer and reverse cross-linked by an overnight incubation in elution buffer at 65 °C. DNA was digested with RNase and Proteinase K, purified, precipitated with phenol chloroform, and eluted in Tris-HCl pH 8.0. Real-time quantitative polymerase chain reaction (qPCR) was performed using *TFF1* oligos: forward: 5′ GGC CAT CTC TCA CTA TGA ATC ACT TCT GCA 3′; and reverse: 5′ GGC AGG CTC TGT TTG CTT AAA GAG CGT TAG 3′.

### Ion torrent

DNA was amplified using Ion AmpliSeq™ Library Kit 2.0 (Life Technologies), then digested, and Ion Xpress™ Barcode adapters ligated and purified with Agencourt AMPure XP magnetic beads (Beckman Coulter). Libraries were quantified by qPCR using an Ion Library Quantification Kit (Life Technologies), templated on the Ion OneTouch2 System (Life Technologies) and sequenced on the Ion PGM System (Life Technologies). Reads were aligned by the PGM server with standard settings to the reference genome hg19; samtools v1.2 was used to calculate the on-target coverage.

IonReporter™ (v4.4) was used for mutation calling (parameters: Data Quality Stringency = 12, Downsample To Coverage = 4000, SNP/InDel/MNP Min Cov Each Strand = 50, SNP/InDel/MNP Min Variant Score = 15, SNP/InDel/MNP Min Coverage = 250, Hotspot Min Variant Score = 6, Hotspot Min Coverage = 150). All mutations called were manually reviewed in IGV and included in the analysis if they had a VAF ≥ 1%.

### Human tumour xenografts modelling relapse on AI therapy

In-vivo studies were carried out in ovariectomized 8- to 12-week-old female BALB/c FOX nude mice in accordance with Home Office guidelines and approved by the Institute of Cancer Research Ethics Committee. MCF72a-LTED tumour xenografts were initiated by the implantation of cells (10^7^) combined with matrigel (1:1) into the left flank. Tumours were established in the absence of E. Once tumours reached a diameter of 7–8 mm, animals were assigned to treatment groups with no statistically significant differences in mean volume before treatment. Animals were treated with either vehicle, fulvestrant administrated subcutaneously weekly (5 mg/kg in olive oil), neratinib (40 mg/kg in 0.5% hydroxypropyl methylcellulose (HPMC)/0.4% Tween 80), or RAD001 (2 mg/kg in 0.5% HPMC/0.4% Tween 80) administered daily by oral gavage for a total of 41 days. Drugs were supplied alone or in the combinations indicated. Tumour growth was assessed weekly in all arms by calliper measurements of the two large diameters. Volumes were calculated according to the formula: a × b^2^ × π/6, where a and b are orthogonal tumour diameters. Tumour volumes were then expressed as mean fold-change in volume at the start of treatment. The study operator was blinded to the treatments.

A second short-term study to address changes in gene expression was performed. Tumours from three mice per treatment were harvested 6 h post-final drug administration following 5 full days of therapy. Tumours were snap frozen in liquid nitrogen for gene expression analysis.

### Immunohistochemical analysis

Tumour fragments were formalin-fixed and paraffin-embedded. Sections were stained for ER using anti-ER antibody (6F11, Novocastra, UK) [17].

### RNA-seq

Libraries were created after the Ribo-zero rRNA removal kit (Illumina) using NEBNext Ultra Directional RNA (NEB) and sequenced using the HiSeq2500 (paired end 100 bp v4 chemistry). Tophat (v2.1) and Cuffdiff (v2.2.1) [18] with default parameters were used for alignment and differential expression analysis. Genes which had a fold-change greater than 50% compared with vehicle in any condition were mapped to KEGG pathway graphs using Pathview [19]. Gene set enrichment analysis (GSEA) [20] was used to identify gene sets that were significantly up- or downregulated in each treatment. [19]. The data supporting this study have been deposited in the NCBI gene expression omnibus (GSE112401).

### Statistical analysis

Statistical analysis was performed using Student’s *t* test or one-way analysis of variance (ANOVA) with Tukey’s to adjust for multiple comparisons. For xenograft studies, overall statistical differences were calculated using the Wilcoxon signed-rank test if the variance was not equal and failed the normality test, otherwise paired *t* tests were used.

## Results

### Effect of RAD001 or neratinib alone or in combination with endocrine therapy on cell growth

**Fig. 2:**
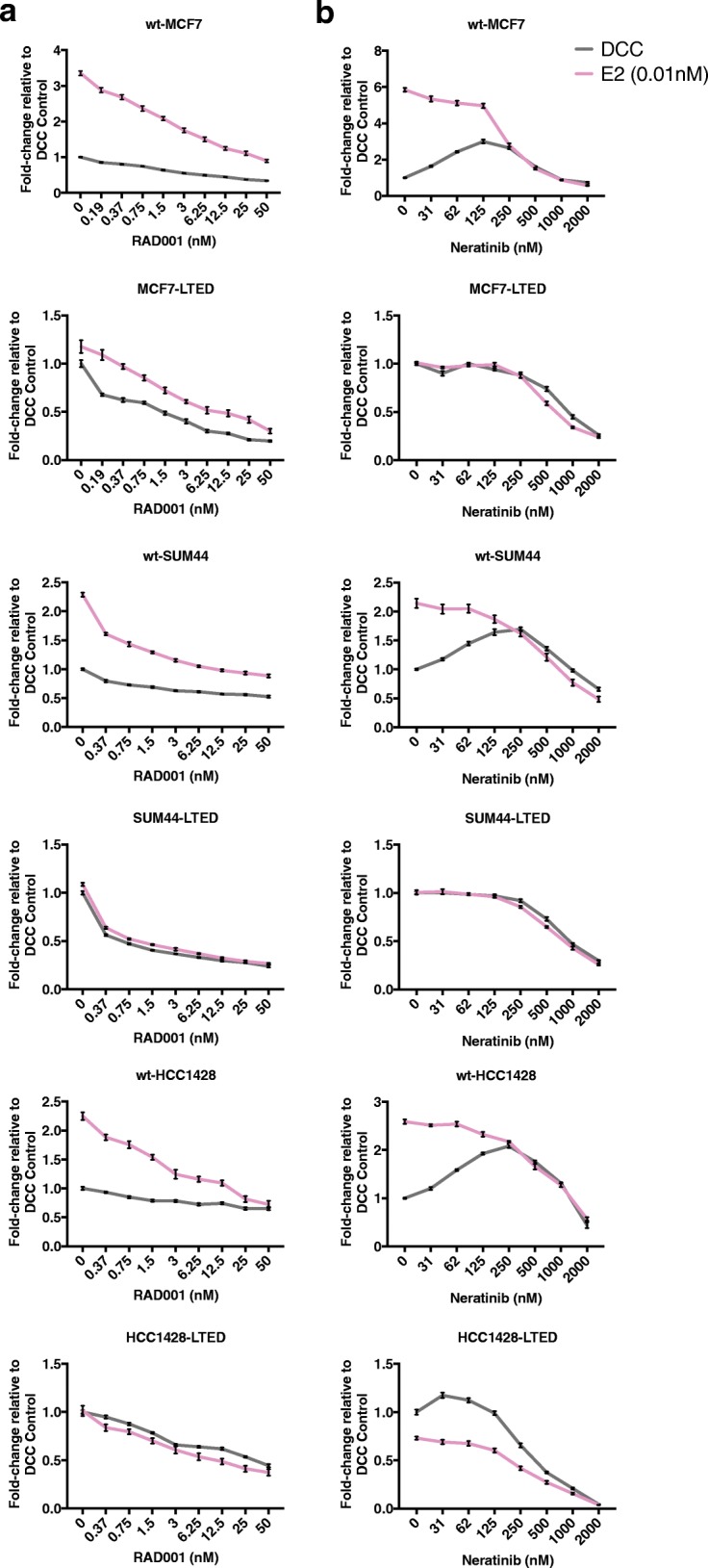
Anti-proliferative effect of **a** RAD001 and **b** neratinib in endocrine-resistant and -sensitive BC cell lines. Cells were treated in the absence or presence of exogenous E2 (0.01 nM) and doubling concentrations of RAD001 or neratinib. Treatments were performed at day 1 and day 3 after seeding. After 6 days of treatment, cell viability was analysed using a cell titer-glo assay. Data are expressed as fold-change relative to dextran charcoal (DCC) control. Error bars represent mean ± SEM

Endocrine-sensitive and LTED BC cell lines retaining ER expression and with varying levels of *EGFR*, *ERBB2*, *ERBB3*, and *FRAP1* expression [21] and differing *PIK3CA*, *ERBB2*, and *ESR1* mutation status (Additional file 1: Figure S1a, b) were assessed for their sensitivity to escalating doses of RAD001 (Fig. 2a) or neratinib (Fig. 2b) in the presence or absence of E2. The addition of RAD001 to wt cell lines in the absence of E2 showed minimal additional anti-proliferative activity compared with E deprivation alone. Contrastingly, in the presence of E2, RAD001 caused a concentration-dependent decrease in proliferation in all wt cell lines tested. Overall, even at the highest concentration of RAD001 (50 nM), the anti-proliferative effect was inferior to that seen with E deprivation alone. Contrastingly, all LTED models showed a concentration-dependent decrease in proliferation in the absence of E2 with varying degrees of sensitivity. It is noteworthy that MCF7-LTED and SUM44-LTED, which harbour an ESR1^Y537S^ mutation, appeared most sensitive with IC_50_ values of 1.5 and 0.5 nM, respectively (Fig. 2a and Additional file 1: Figure S1c).

Escalating concentrations of neratinib caused a hormetic (bell shaped) proliferation curve in all the wt cell lines tested in the absence of E2, with mid-range doses causing an approximate two- to threefold increase in proliferation. IC_50_ values for neratinib were not achieved in this setting (Fig. 2b and Additional file 1: Figure S1d). LTED derivatives in the absence of E2 showed IC_50_ values of 900 nM for MCF7-LTED and SUM44-LTED and 400 nM for HCC1428-LTED. The addition of E2 increased the sensitivity of all wt cell lines, with wt-MCF7 having the lowest recorded IC_50_ (300 nM) (Fig. 2b and Additional file 1: Figure S1d).

We subsequently assessed the interaction between RAD001 or neratinib with escalating doses of 4-OHT and ICI (Additional file 2: Figure S2 and Additional file 3: Figure S3). In the presence of exogenous E2, ICI and 4-OHT caused a concentration-dependent decrease in proliferation in all wt and LTED cells. For all cell lines tested, RAD001 enhanced the sensitivity to 4-OHT and ICI with the exception of the HCC1428-LTED, in which no further anti-proliferative effect was detected when RAD001 was combined with 4-OHT (Additional file 2: Figure S2b). Similar responses were observed when neratinib was combined with 4-OHT or ICI, with the exception of wt-HCC1428 with ICI and HCC1428-LTED with 4-OHT in which neratinib showed minimal impact, particularly at higher concentrations (> 1 nM) (Additional file 3: Figure S3a, b).

### Dual blockade of mTORC1 and ERBB signalling in combination with endocrine therapy enhances anti-proliferative effectiveness

**Fig. 3:**
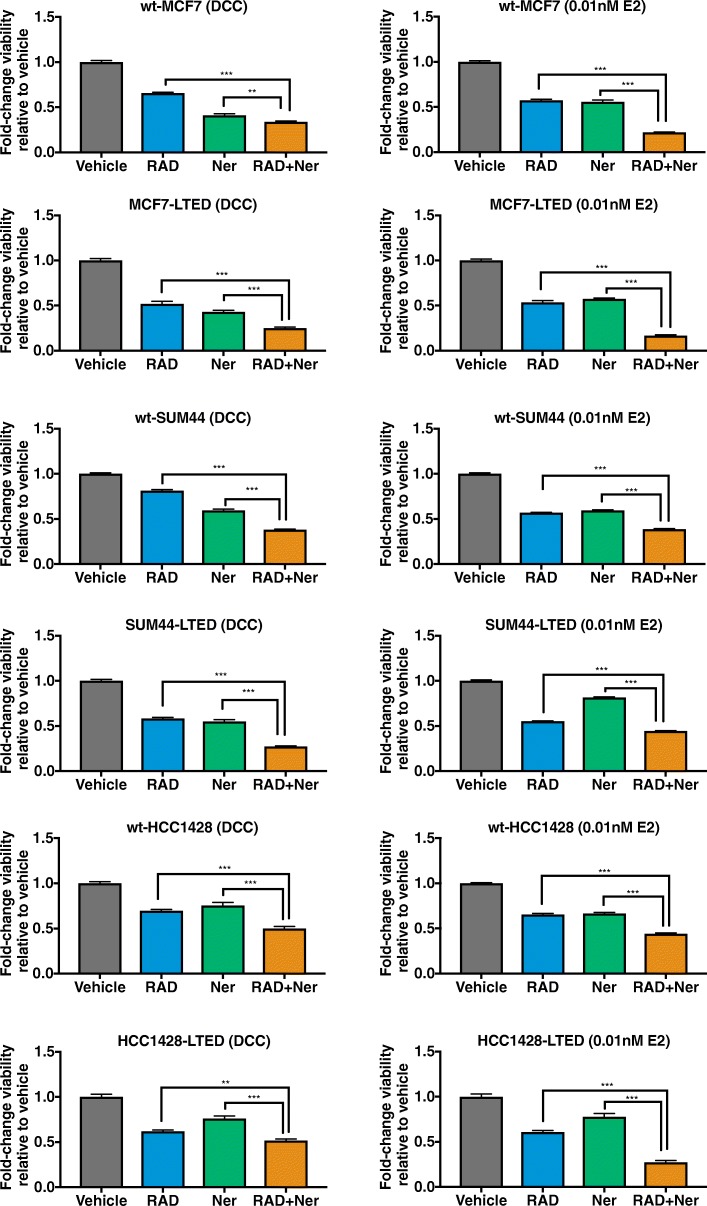
Anti-proliferative effect of RAD001 (RAD), neratinib (Ner), or their combination in endocrine-resistant and -sensitive BC cell lines. Cell lines were treated with vehicle or sub-optimal concentrations for each drug alone or in combination, both in the absence and presence of 0.01 nM exogenous E2. After 6 days of treatment, cell viability was analysed using cell titer-glo and data expressed as fold-change relative to vehicle control. Error bars represent mean ± SEM. *p < 0.05; **p < 0.01; ***p < 0.001. Concentrations used in dextran charcoal (DCC): wt-MCF7 (0.75 nM RAD001, 2000 nM neratinib); MCF7-LTED (0.75 nM RAD001, 500 nM neratinib); wt-SUM44 (0.75 nM RAD001, 2000 nM neratinib); SUM44-LTED (0.4 nM RAD001, 500 nM neratinib); wt-HCC1428 (12.5 nM RAD001, 1200 nM neratinib); HCC1428-LTED (3 nM RAD001, 250 nM neratinib). Concentrations used in E2: wt-MCF7 (1.5 nM RAD001, 200 nM neratinib); MCF7-LTED (1.5 nM RAD001, 300 nM neratinib); wt-SUM44 (0.37 nM RAD001, 450 nM neratinib); SUM44-LTED (0.37 nM RAD001, 250 nM neratinib); wt-HCC1428 (1.5 nM RAD001, 500 nM neratinib); HCC1428-LTED (3 nM RAD001, 250 nM neratinib)

As altered growth factor signalling has been associated with mTORC1 blockade providing a route of resistance to long-term inhibition of this kinase [9], we examined the strategy of combining RAD001 with neratinib in the presence of continued endocrine therapy. To assess this, sub-optimal concentrations of each agent were combined in the presence or absence of E2. For all the cells lines tested, both in the presence and absence of E2, the combination of RAD001 and neratinib showed a superior anti-proliferative effect compared with either agent alone (Fig. 3).

To assess the effect of combining mTORC1 and ERBB suppression with endocrine therapy, cell lines were treated with sub-optimal concentrations of RAD001 or neratinib alone or in combination, with escalating doses of 4-OHT or ICI. The combination of RAD001 and neratinib enhanced the efficacy of both endocrine agents, particularly at the lower concentration range (Additional file 4: Figure S4a, b).

### Effect of the combination of RAD001 and neratinib on cell signalling

**Fig. 4:**
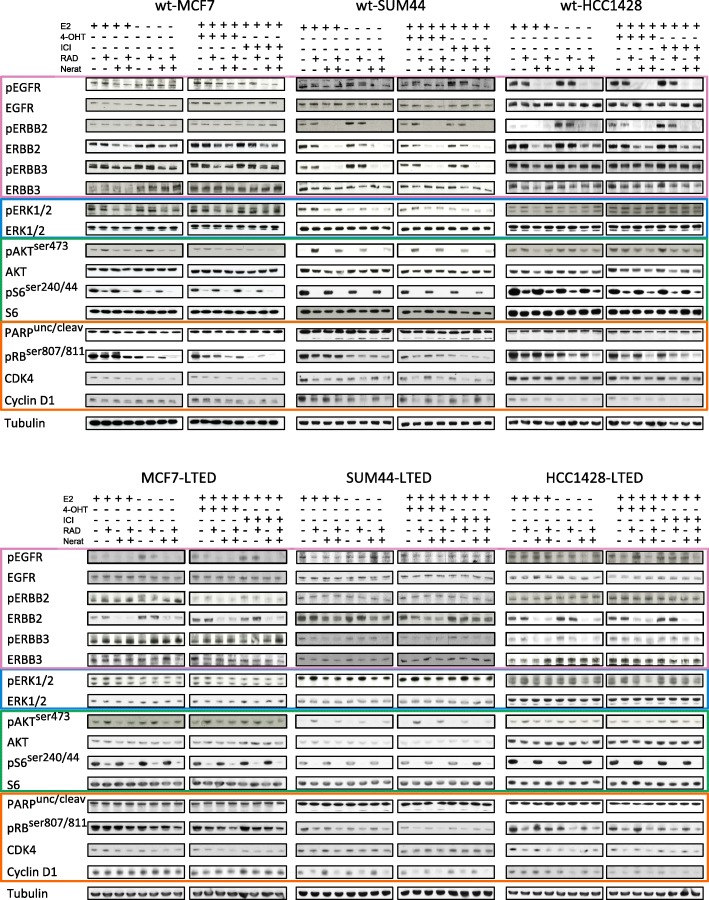
Effect of RAD001 (RAD), neratinib (Ner), or their combination with endocrine agents on cell signalling pathways governing cell cycle. Endocrine-resistant and -sensitive BC cell lines were treated for 24 h with the drug combinations indicated. Whole-cell extracts were assessed for expression on S6 kinase, ERK1/2, AKT, and ERBB signalling together with markers of cell cycle and apoptosis by immunoblotting. IC_50_ values were used for RAD001 and neratinib together with standard concentrations of oestradiol (E2; 0.01 nM), 4-hydroxytamoxifen (4-OHT; 10 nM), and fulvestrant (ICI; 1 nM) (with exception of HCC1428-LTED where 10 nM was used). ERBB pathways are highlighted in pink, ERK1/2 in blue, mTORC1/AKT in green, and cell cycle in orange. wt-MCF7 (2 nM RAD001, 500 nM neratinib); MCF7-LTED (4 nM RAD001, 750 nM neratinib); wt-SUM44 (3 nM RAD001, 700 nM neratinib); SUM44-LTED (3 nM RAD001, 700 nM neratinib); wt-HCC1428 (3 nM RAD001, 1000 nM neratinib); HCC1428-LTED (10 nM RAD001, 500 nM neratinib)

To investigate the effect of RAD001 and neratinib alone or in combination with endocrine agents on cellular signal transduction pathways, parental (endocrine-sensitive) and LTED cell lines were treated with the drug combinations indicated for 24 h ± E2, 4-OHT, or ICI (Fig. 4). As expected, phosphorylation of S6 was dramatically suppressed by RAD001 alone or in combination with neratinib in all cell lines tested. Contrastingly, neratinib caused cell line-specific effects on members of the ERBB family. For instance, neratinib caused a significant downregulation in total ERBB2 in all cell lines and reduced phosphorylated epidermal growth factor receptor (EGFR) in MCF7-LTED, wt-SUM44, and wt-HCC1428, as well as phosphorylated ERBB3 in wt-MCF7, wt-SUM44, and HCC1428-LTED. Furthermore, RAD001 caused an upregulation of phosphorylated AKT in all cell lines tested and increased phosphorylation of ERK1/2 in wt-SUM44, and to a lesser degree in SUM44-LTED, wt-HCC1428, and HCC1428-LTED, indicative of rapid re-wiring previously associated with resistance to mTORC1 inhibition [8–10]. It is noteworthy that, in the majority of cell lines, the combination of RAD001 with neratinib suppressed the upregulation of phosphorylated AKT and ERK1/2 (Fig. 4).

To investigate the impact of RAD001 or neratinib combined with 4-OHT or ICI versus the triple combination on cell cycle progression, we assessed the abundance of pertinent cell cycle proteins. The combination of endocrine therapy with either RAD001 or neratinib decreased levels of phosphorylated RB, Cyclin D1, and CDK4 to a greater extent than endocrine therapies alone. However, as expected, the greatest degree of inhibition was evident with triple combination concomitantly blocking mTORC1, ERBB, and ER signalling, an effect most evident with ICI (Fig. 4). No substantial increase in cleaved PARP was evident, suggesting minimal impact on apoptosis.

### Effect of RAD001 alone or in combination with neratinib on ER transactivation

**Fig. 5:**
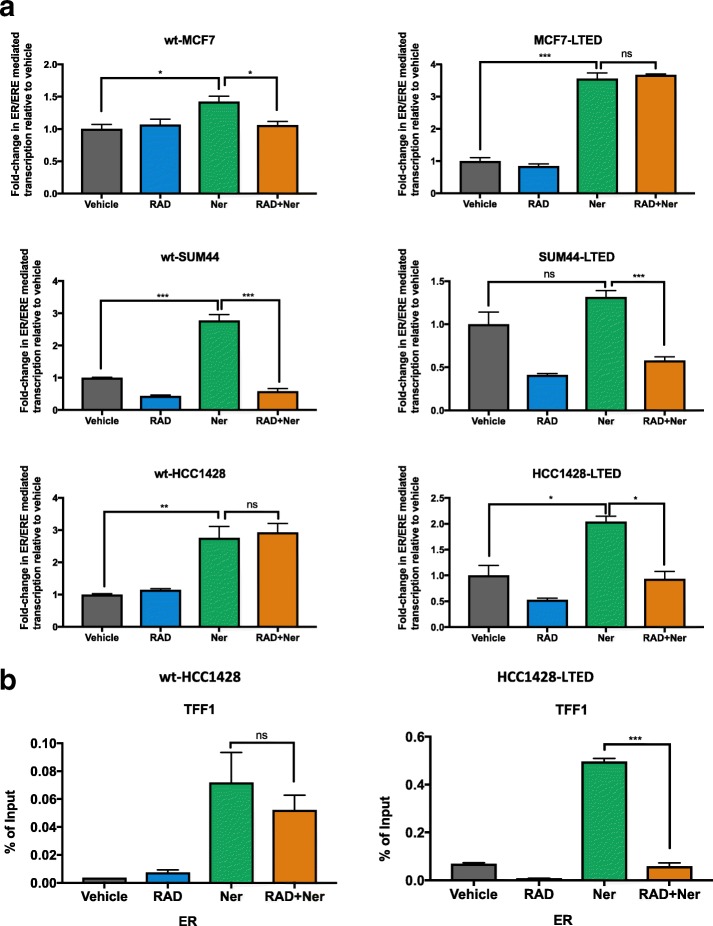
Effect of RAD001 (RAD), neratinib (Ner), or their combination on oestrogen receptor (ER)-mediated transactivation and recruitment of the ER basal transcription machinery. **a** Cell lines were co-transfected with EREIItkLuc and pCH110 and treated for 24 h with RAD001 and neratinib in the absence of E2 (DCC). Luciferase activity was normalized by β-galactosidase from triplicate wells and fold-changes expressed relative to the DCC control. **b** ChIP analysis to determine the effect of neratinib, RAD001, or the combination on recruitment of ER to the *TFF1* promoter in wt-HCC1428 and HCC1428-LTED. Error bars represent mean ± SEM. *p < 0.05; **p < 0.01; ***p < 0.001. Concentration used for transactivation assay and ChIP: wt-MCF7 (2 nM RAD001, 500 nM neratinib); MCF7-LTED (4 nM RAD001, 650 nM neratinib); wt-SUM44 (3 nM RAD001, 700 nM neratinib); SUM44-LTED (3 nM RAD001, 700 nM neratinib); wt-HCC1428 (3 nM RAD001, 700 nM neratinib); HCC1428-LTED (10 nM RAD001, 300 nM neratinib). ns not significant

The majority of the patients who relapse on endocrine therapy retain expression of ER. In-vitro data have shown that ER can be phosphorylated in a ligand-independent manner, circumventing the need for steroid hormones. Major pathways associated with this include ERBB/ERK1/2 and PI3K/AKT/mTOR [6]. To assess whether interactions between the drugs impacted on E-independent transactivation, endocrine-sensitive and LTED cell lines were transiently transfected with an ERE-luciferase reporter construct and treated with either RAD001, neratinib, or the combination with or without E2, 4-OHT, or ICI (Fig. 5a and Additional file 5: Figure S5). Under E-deprived (DCC) conditions, mimicking the effects of an AI, neratinib caused a significant enhancement in ER/ERE-mediated transcription compared with the vehicle control in all cell lines tested (*p* ≤ 0.03), with the exception of SUM44-LTED which showed a trend to significance (*p* = 0.1). RAD001 alone suppressed ER-mediated transcription to varying extents across the cell lines. Most notably, wt-SUM44 and SUM44-LTED together with HCC1428-LTED appeared most sensitive, with a drop in ER-mediated transcription of approximately 50% compared with the vehicle control. In contrast, wt-MCF7 and their LTED derivatives, as well as wt-HCC1428, were unaffected. Of note, the combination of RAD001 and neratinib appeared to negate the neratinib-driven increase in ER-mediated transcription in several of the models (*p* ≤ 0.03). However, transactivation remained higher than that seen with RAD001 alone, and indeed the combination did not reduce the effect of neratinib in wt-HCC1428 or MCF7-LTED.

To address the enhanced ER/ERE-mediated transactivation in response to neratinib, chromatin immunoprecipitation was performed in wt-HCC1428 and HCC1428-LTED cells, which showed differential responses to neratinib when combined with RAD001 (Fig. 5a). ChIP analysis of ER recruitment in wt-HCC1428 showed enrichment at the *TFF1* promoter in response to neratinib compared with the vehicle control. The combination of RAD001 and neratinib had no significant impact on recruitment, in keeping with the ER/ERE-mediated transcription analysis (Fig. 5a, b). Contrastingly, HCC1428-LTED showed enhanced recruitment of ER in response to neratinib, which was significantly reduced by the addition of RAD001 (*p* < 0.001) (Fig. 5b), suggesting that context-specific impacts on ER-mediated transcription were responsible for these events.

Sub-optimal concentrations of 4-OHT or ICI caused a 40–60% reduction in ER-transactivation in all cell lines, with the exception of HCC1428-LTED in response to 4-OHT where the reduction did not meet statistical significance. Similarly, SUM44-LTED, which harbours a Y537S mutation in *ESR1*, showed no response to either 4-OHT or ICI. The combination of RAD001 or neratinib with endocrine therapy showed no further reduction in ER-mediated transcription compared with endocrine therapy alone in all cell lines tested with the exception of the SUM44 models. In this setting, RAD001 in combination with 4-OHT or ICI caused a significant reduction in ER-mediated transcription. However, the addition of neratinib showed no impact and, indeed, the triple combination impeded ER-mediated transactivation to a similar degree as RAD001 when combined with either endocrine agent. This suggests that wt-SUM44 and SUM44-LTED are particularly sensitive to cross-talk between ER and mTORC1 signalling. Indeed, the combination of 4-OHT with RAD001 significantly reduced pER^ser167^ and total ER (Additional file 5: Figure S5).

### Effect of RAD001 alone or in combination with neratinib and/or fulvestrant in vivo

**Fig. 6:**
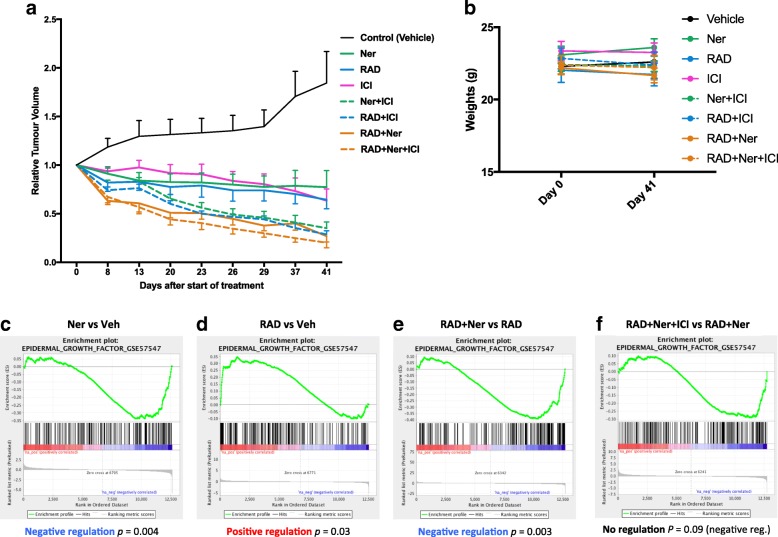
Effect of RAD001 (RAD) and neratinib (Ner) alone or in combination with endocrine therapy in vivo. **a** Long-term study assessing the relative mean changes in tumour volume over 41 days of treatment and **b** effect of drug regimes on animal weight. Error bars represent mean ± SEM (*n* = 7–9 animals per group). RAD001, 2 mg/kg; neratinib, 40 mg/kg; fulvestrant (ICI), 5 mg/kg. **c–f** GSEA enrichment plots for 198 genes known to be induced by sustained activation of ERK in response to EGF activity. Plots show the profile of the running Enrichment Score and positions of GeneSet Members on the Rank Ordered List for rank gene lists generated from the comparison of **c** neratinib vs. vehicle (Veh), **d** RAD001 vs. vehicle, **e** RAD001 + neratinib vs. RAD001, and **f** RAD001 + neratinib + ICI vs. RAD001 + neratinib

To assess the effect of the drugs as monotherapies or combinations on tumour volume in vivo, mice were implanted with MCF72a-LTED ER^+^ tumour cells, which grow independently of exogenous E and model relapse on an AI (details regarding generation of this model are shown in Additional file 6: Figure S6a). Animals were treated with vehicle, monotherapy (RAD001, neratinib or fulvestrant), dual, or triple therapy combinations. The mean fold-change in tumour volume for each treatment was expressed relative to the start of treatment (Fig. 6a). Tumour volumes for the control vehicle group increased 1.8-times over the treatment period (*p* = 0.1). All monotherapies caused a reduction in tumour volume by day 41 compared with the start of treatment (RAD001: 36%, *p* = 0.03; neratinib: 23%, *p* = 0.6; fulvestrant: 37%, p = 0.03). Dual combination therapies showed a further reduction in tumour volume (RAD001 + neratinib: 73%, *p* = 0.03; RAD001 + fulvestrant: 72%, *p* = 0.004; neratinib + fulvestrant: 65%, *p* = 0.004). Triple combination of RAD001, neratinib, plus fulvestrant was the most effective, resulting in an 80% inhibition in tumour growth (*p* = 0.008). Assessment of mouse weights showed that the drug combinations had no significant effect during the course of the study (Fig. 6b).

To assess dynamic changes in gene expression in response to RAD001, neratinib, or the combinations with fulvestrant, a second short-term xenograft study was carried out followed by RNA-seq. Differentially expressed genes were subjected to pathway analysis (Additional file 6: Figure S6b). As expected, RAD001 increased *AKT* and *ERK* expression, which was reduced by the addition of neratinib. *ER* expression was elevated with neratinib treatment compared with RAD001 and fulvestrant. Notably, *CoR* expression was elevated in the dual (RAD001 plus neratinib) and triple combination. Furthermore, global effects on proliferation showed a greater reduction with the dual and triple combinations compared with single agents. This was further supported by the assessment of *E2F* target genes (Additional file 6: Figure S6c). As expected, the triple combination of RAD001, neratinib, and fulvestrant suppressed the expression of cell cycle-associated genes (*CCNE1*, *CCNL1*, *CDK3*, *CDK7*, and *CDK9*) when compared with RAD001 alone or in combination with neratinib, in keeping with the longer-term xenograft study (Additional file 7: Figure S7a). Based on the pathway analysis, we used GSEA to assess the dynamic changes in EGFR/ERBB2 signalling after blockade with neratinib, RAD001, or the combinations. Neratinib reduced the expression of genes associated with EGF/EGFR activation of ERK signalling [22] (*p* = 0.004) (Fig. 6c); contrastingly, RAD001 significantly induced expression of this gene set (*p* = 0.03) (Fig. 6d), an observation in support of rapid re-wiring associated with resistance to mTORC1 inhibition. As expected, the combination of neratinib and RAD001 significantly reduced expression of the EGFR/ERK gene set (*p* = 0.003) (Fig. 6e). The addition of fulvestrant to the double combination showed a further trend in the reduction of this (*p* = 0.09) (Fig. 6f). Finally, assessment of fulvestrant alone or in combination with RAD001 showed no impact on EGFR/EGF-regulated genes; however, the addition of neratinib significantly reduced the EGFR/ERK gene set (*p* < 0.0001) (Additional file 7: Figure S7b). Taken together, this suggests that the addition of neratinib negates the EGF/EGFR feedback loop, providing further support for the anti-proliferative effect seen with the triple combination and highlighting the potential utility of concomitantly targeting three cellular signalling nodes.

## Discussion

In-vitro and in-vivo analysis of tumours that are resistant to endocrine therapy suggests complex interplay between cell signalling molecules, which cooperate to govern escape mechanisms. Treatment with small molecule inhibitors of pertinent pathways may provide clinical benefit. For instance, recent studies have shown that blockade of mTORC1 signalling in combination with AI therapy causes a marked increase in PFS in patients with metastatic ER^+^ BC (BOLERO-2) [7]; however, relapse remains a significant clinical issue.

To identify potential pathways attributed to the lack of response to RAD001, we previously carried out a molecular study in cell lines adapted to LTED, modelling the patient cohort on the BOLERO-2 study. We showed that RAD001 induced a feedback loop via ERBB2/3, which could potentiate resistance [9]. In further support of this, Carracedo and colleagues [10] showed a similar upregulation of ERK1/2 in response to mTORC1 inhibition. Furthermore, studies using ERBB inhibitors have highlighted resistance pathways via upregulation of PI3K/mTORC/AKT signalling [23, 24], suggesting a high degree of cross-talk between these two pivotal cellular signal transduction pathways. In addition, both PI3K/mTORC/AKT and ERK1/2 have been implicated in the ligand-independent activation of ER, leading to resistance to endocrine therapy [6]. Based upon these observation, we hypothesised that simultaneous blockade of all three cellular nodes may provide potential benefit in circumventing the resistance seen with individual therapies. To test this hypothesis, we assessed the combination of neratinib, a pan-ERBB inhibitor, with the mTORC1 inhibitor, RAD001, in the presence of various endocrine agents in models mimicking endocrine-sensitive and AI-resistant disease.

Surprisingly, treatment of endocrine-sensitive BC cells with neratinib in the absence of exogenous E2 generated a hormetic response curve, with a lower concentration of the drug causing a marked increase in proliferation and associated ER-mediated transactivation. Previous clinical studies have reported a mixed benefit of the combination of AI with EGFR or ERBB2 blockade in primary or naive advanced BC, and in some cases have shown a trend towards poorer outcome [25, 26]. Furthermore, this observation is not only evident with targeted EGFR and ERBB2 RTKs but also with pan-ERBB inhibitors, such as AZD8931, in which a recent phase II randomised study in combination with an AI in women with endocrine-naive advanced BC provided no benefit compared with anastrozole alone and did not delay endocrine resistance in this patient population [27]. Notably, treatment of LTED cell lines also showed enhanced ER/ERE-mediated transcription and recruitment of ER to target promoters in response to neratinib. However, in contrast to the parental cell lines, proliferation decreased. The decrease in proliferation is in keeping with clinical studies, which suggest that patients who have acquired resistance to endocrine therapy via upregulation of EGFR/ERBB2 may benefit from pan-ERBB inhibition, as they become more reliant on growth factor signalling as the mitogenic driver [26].

In contrast to E deprivation, the combination of neratinib with 4-OHT or ICI showed an enhanced anti-proliferative effect in the majority of parental cell lines. However, although the combination outperformed either treatment alone at the concentrations tested, the magnitude of benefit was less than would be expected from an additive benefit from either treatment alone. These data are in keeping with previous in-vitro, as well as clinical, studies assessing the combination of EGFR blockade with gefitinib to delay the onset of endocrine resistance [28, 29]. The mechanism underlying this remains unclear, but in-vitro studies suggest that tamoxifen-bound ER binds co-repressor molecules allowing the ERBB2 promoter to sequester SRC1 and AIB1, leading to transcription of ERBB2 and potentially providing the target for RTK inhibition [30].

Treatment with RAD001 showed differential effects on cell proliferation. Most notably, the LTED derivatives showed lower IC_50_ values compared with their parental cell lines with the exception of HCC1428. *PIK3CA* mutation status was not a governing factor of sensitivity, as both SUM44 and HCC1428 harbour the wt gene. Wt-SUM44 in the absence of exogenous E2 showed no response to RAD001; however, this was attributed to the fact that, under E-deprived conditions, the majority of cells are in cell cycle arrest and as such further perturbation provides little effect. This was confirmed by the observation in the presence of E2 where the IC_50_ was approximately 3 nM, similar to that seen in MCF7-LTED. These data again show that mutation status is not the governing feature of sensitivity, and that cellular context remains more informative, in keeping with the translational study of BOLERO-2 which showed that *PIK3CA* mutations were not in themselves predictive of clinical benefit to mTORC1 inhibitors [31]. Treatment with RAD001 decreased ER-mediated transcription as a result of reduced S6 kinase activity and subsequent phosphorylation of ER^ser167^, which was particularly notable in wt-SUM44 and their LTED derivative. Allosteric inhibition of mTORC1 led to an increase in phosphorylated AKT, indicative of the previously observed S6 feedback loop [8]. Furthermore, in certain cell lines, ERK1/2 was also elevated. This may indicate that phospho-ERK activation following mTORC1 inhibition occurs via cross-talk with the PI3K-RAS signalling pathway [10]. In selected cell lines, evidence suggested that enhanced ERBB signalling may be responsible for the observed ERK activation. Indeed, GSEA analysis showed that RAD001 increased expression of EGF/EGFR-associated genes which was significantly suppressed by the addition of neratinib. In keeping with this, our in-vivo study showed concordant data in which the triple combination significantly reduced tumour volume.

Taken together, these data support the combination of mTORC1 blockade with inhibition of ERBB signalling and ER function in ER^+^ BC, highlighting the potential clinical utility. Further support for the dual blockade of both mTORC1 and ERBB signalling comes from a recent phase I clinical trial piloting the combination of neratinib with temsirolimus, in which antitumoral activity in patients with advanced BC was evident [32].

## Conclusions

In conclusion, our results provide support for the combination of RAD001 together with neratinib and endocrine therapy to re-sensitise endocrine-resistant tumours to the anti-proliferative effects of endocrine therapy. Most notably, the combination with ICI, disabling both the ER and AKT axis, appeared superior. Furthermore, even within this restricted panel of cell lines, the heterogeneity of response highlights the need to identify common adaptive nodes.

## Additional files

Additional file 1: **Figure S1.** IC_50_ values for the anti-proliferative effect of RAD001 and neratinib in relation to the *ESR1*, *ERBB2*, and *PIK3CA* mutational status in endocrine-resistant and -sensitive BC cell lines. (a) Mutational or wt status is depicted in grey and white, respectively, for *ESR1*, *ERBB2*, and *PIK3CA*. (b) Varying degrees of expression of genes encoding proteins targeted by fulvestrant, neratinib, and RAD001 showing heterogeneity in the cell lines tested. (c,d) Cells were treated in the absence or presence of exogenous oestradiol (E2) (0.01 nM) and doubling concentrations of (c) RAD001 or (d) neratinib. Treatments were performed at day 1 and day 3 after seeding. After 6 days of treatment, cell viability was analysed using a cell titer-glo assay and IC_50_ values were plotted. (PDF 156 kb)

Additional file 2: **Figure S2.** Anti-proliferative effect of RAD001 in combination with endocrine agents (a) 4-OHT and (b) ICI. Endocrine-resistant and -sensitive BC cell lines were treated with a combination of RAD001 (3 nM) and increasing concentrations of (a) 4-OHT or (b) ICI for 6 days with media change at day 3. Cell viability was analysed using a cell titer-glo assay. Data are expressed as fold-change relative to vehicle control. Error bars represent mean ± SEM. (PDF 196 kb)

Additional file 3: **Figure S3.** Anti-proliferative effect of neratinib in combination with endocrine agents (a) 4-OHT and (b) ICI. Endocrine-resistant and -sensitive BC cell lines were treated with a combination of neratinib (500 nM in wt-MCF7 and MCF7-LTED; 700 nM in wt-SUM44, SUM44-LTED, and wt-HCC1428; 300 nM in HCC1428-LTED) and increasing concentrations of (a) 4-OHT or (b) ICI for 6 days with media change at day 3. Cell viability was analysed using a cell titer-glo assay. Data are expressed as fold-change relative to vehicle control. Error bars represent mean ± SEM. (PDF 195 kb)

Additional file 4: **Figure S4.** Anti-proliferative effect combination of RAD001 and neratinib together with endocrine agents (a) 4-OHT and (b) ICI. Endocrine-resistant and -sensitive BC cell lines were treated with a combination of RAD001 and neratinib and increasing concentrations of (a) 4-OHT or (b) ICI for 6 days with media change at day 3. Cell viability was analysed using a cell titer-glo assay. Data are expressed as fold-change relative to vehicle control. Error bars represent mean ± SEM. wt-MCF7 (1.5 nM RAD001; 200 nM neratinib); MCF7-LTED (1.5 nM RAD001; 300 nM neratinib); wt-SUM44 (0.37 nM RAD001; 450 nM neratinib); SUM44-LTED (0.37 nM RAD001; 250 nM neratinib); wt-HCC1428 (1.5 nM RAD001; 500 nM neratinib); HCC1428-LTED (3 nM RAD001; 250 nM neratinib). (PDF 208 kb)

Additional file 5: **Figure S5.** Effect of RAD001, neratinib, or their combination with endocrine agents on ER-mediated transactivation and ER signalling. Cell lines were co-transfected with EREIItkLuc and pCH110, and treated for 24 h with the drug combinations indicated. IC_50_ values were used for RAD001 and neratinib together with standard concentrations of E2 (0.01 nM), 4-OHT (0.1 nM), and ICI (0.1 nM). Luciferase activity was normalized by β-galactosidase from triplicate wells and fold-changes expressed relative to the E2 control. Error bars represent mean ± SEM. *p < 0.05; **p < 0.01; ***p < 0.001. wt-MCF7 (2 nM RAD001; 500 nM neratinib); MCF7-LTED (4 nM RAD001; 650 nM neratinib); wt-SUM44 (3 nM RAD001; 700 nM neratinib); SUM44-LTED (3 nM RAD001; 700 nM neratinib); wt-HCC1428 (3 nM RAD001; 700 nM neratinib); HCC1428-LTED (10 nM RAD001; 300 nM neratinib). Western blot was used to assess changes in phosphorylation of the ER in response to RAD001, neratinib, or their combination together with endocrine agents. IC_50_ values were used for RAD001 and neratinib together with standard concentrations of E2 (0.01 nM), 4-OHT (10 nM), and ICI (1 nM) (with the exception of HCC1428-LTED where 10 nM was used). wt-MCF7 (2 nM RAD001; 500 nM neratinib); MCF7-LTED (4 nM RAD001; 750 nM neratinib); wt-SUM44 (3 nM RAD001; 700 nM neratinib); SUM44-LTED (3 nM RAD001; 700 nM neratinib); wt-HCC1428 (3 nM RAD001; 1000 nM neratinib); HCC1428-LTED (10 nM RAD001; 500 nM neratinib). (PDF 1240 kb)

Additional file 6: **Figure S6.** Assessment of dynamic changes in gene expression in response to RAD001, neratinib, or the combinations with fulvestrant. (a) MCF72a cells, which were previously engineered to express aromatase (*CYP19*) [33] were implanted into ovariectomised mice under androstenedione support. In this setting, MCF72a cells convert androstenedione in to oestrogen to drive proliferation. Once tumours developed, androstenedione was withdrawn. After a lag phase, tumour growth occurred synonymous with ligand independence. Assessment of the MCF72a-LTED showed continued expression of ER and proliferation in the absence of exogenous oestrogen providing a model of AI relapse. (b) Changes to gene expression (log2 difference drug – vehicle) as detected by RNA-seq for five drug combinations (neratinib, RAD001, ICI, neratinib + RAD001, and neratinib + RAD001 + ICI) were mapped to KEGG pathway graphs using Pathview (https://bioconductor.org/packages/release/bioc/html/pathview.html). Genes with a fold-change greater than 50% compared with vehicle in any condition were selected in order to expand the list of differentially expressed genes, allowing the identification of subtle changes in gene expression; for example, kinases or transcription factors that might have significant impact on downstream gene expression. A heatmap for each gene in shown. (c) Assessment of expression of E2F target genes in response to neratinib and RAD001. (PDF 1861 kb)

Additional file 7: **Figure S7.** Assessment of dynamic changes in expression of cell cycle regulatory genes. (a) Log_2_ differences in *CCNE1*, *CCNL1*, *CDK3*, *CDK7*, and *CDK9* gene expression following treatment with RAD001, RAD001 + neratinib, and RAD001 + neratinib + fulvestrant (ICI), compared with vehicle. (b) GSEA enrichment plots for 198 genes known to be induced by sustained activation of ERK in response to EGF activity. Plots show the profile of the running Enrichment Score and positions of GeneSet Members on the Rank Ordered List for rank gene lists generated from the comparison of: ICI vs. vehicle; neratinib + ICI vs. ICI; RAD001 + ICI vs. ICI; and RAD001 + neratinib + ICI vs. RAD001 + ICI. (PDF 721 kb)

## Abbreviations

4-OHT: 4-Hydroxytamoxifen
AI: Aromatase inhibitors
BC: Breast cancer
DCC: Dextran charcoal
E: Oestrogen
E2: Oestradiol
ER: Oestrogen receptor
FBS: Fetal bovine serum
GSEA: Gene set enrichment analysis
ICI: Fulvestrant
LTED: Long-term oestrogen deprivation
PFS: Progression-free survival
RTK: Receptor tyrosine kinase
