# Supplementary figures and images for: Targeting tumour re-wiring by triple blockade of mTORC1, epidermal growth factor, and oestrogen receptor signalling pathways in endocrine-resistant breast cancer
Source: Breast Cancer Res. 2018 Jun 8;20:44. doi: 10.1186/s13058-018-0983-1 (PMC5992820; doi:10.1186/s13058-018-0983-1)

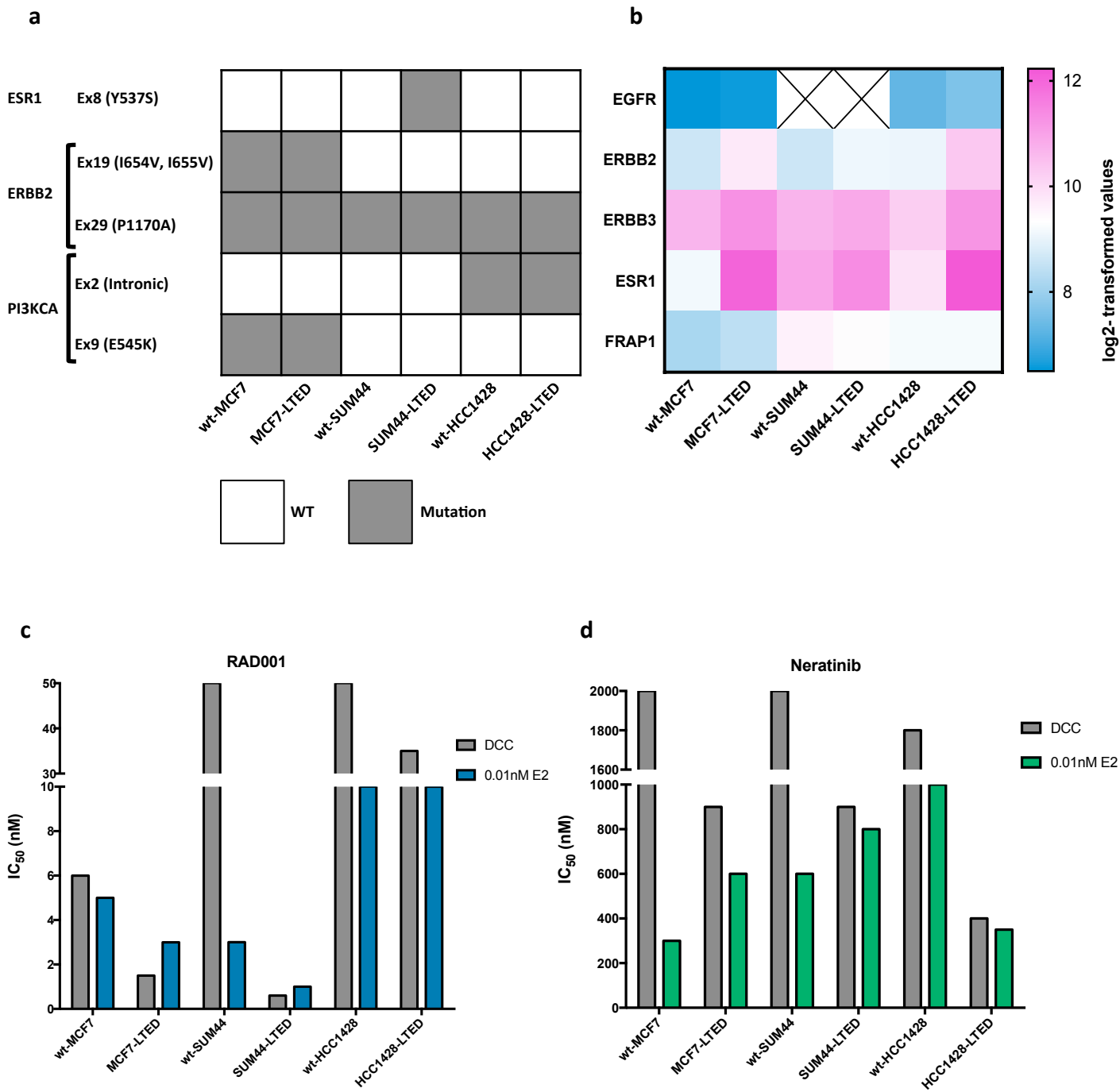

Figure S1

Supplement: Supplementary file 1 — Figure S1. IC50 values for the anti-proliferative effect of RAD001 and neratinib in relation to the ESR1, ERBB2, and PIK3CA mutational status in endocrine-resistant and -sensitive BC cell lines. (a) Mutational or wt status is depicted in grey and white, respectively, for ESR1, ERBB2, and PIK3CA. (b) Varying degrees of expression of genes encoding proteins targeted by fulvestrant, neratinib, and RAD001 showing heterogeneity in the cell lines tested. (c,d) Cells were treated in the absence or presence of exogenous oestradiol (E2) (0.01 nM) and doubling concentrations of (c) RAD001 or (d) neratinib. Treatments were performed at day 1 and day 3 after seeding. After 6 days of treatment, cell viability was analysed using a cell titer-glo assay and IC50 values were plotted. (PDF 156 kb) [file 13058_2018_983_MOESM1_ESM.pdf]

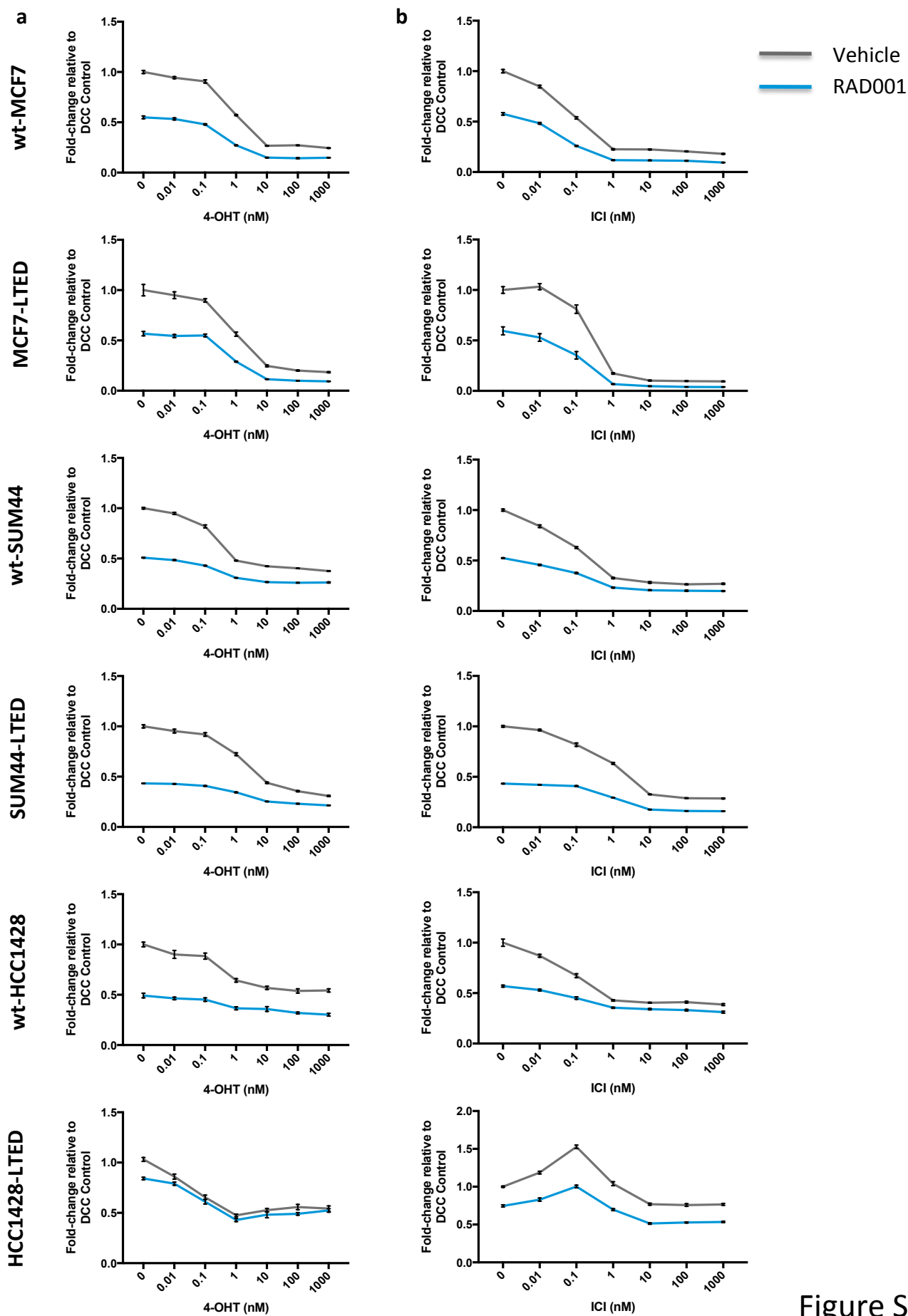

Figure S2

Supplement: Supplementary file 2 — Figure S2. Anti-proliferative effect of RAD001 in combination with endocrine agents (a) 4-OHT and (b) ICI. Endocrine-resistant and -sensitive BC cell lines were treated with a combination of RAD001 (3 nM) and increasing concentrations of (a) 4-OHT or (b) ICI for 6 days with media change at day 3. Cell viability was analysed using a cell titer-glo assay. Data are expressed as fold-change relative to vehicle control. Error bars represent mean ± SEM. (PDF 196 kb) [file 13058_2018_983_MOESM2_ESM.pdf]

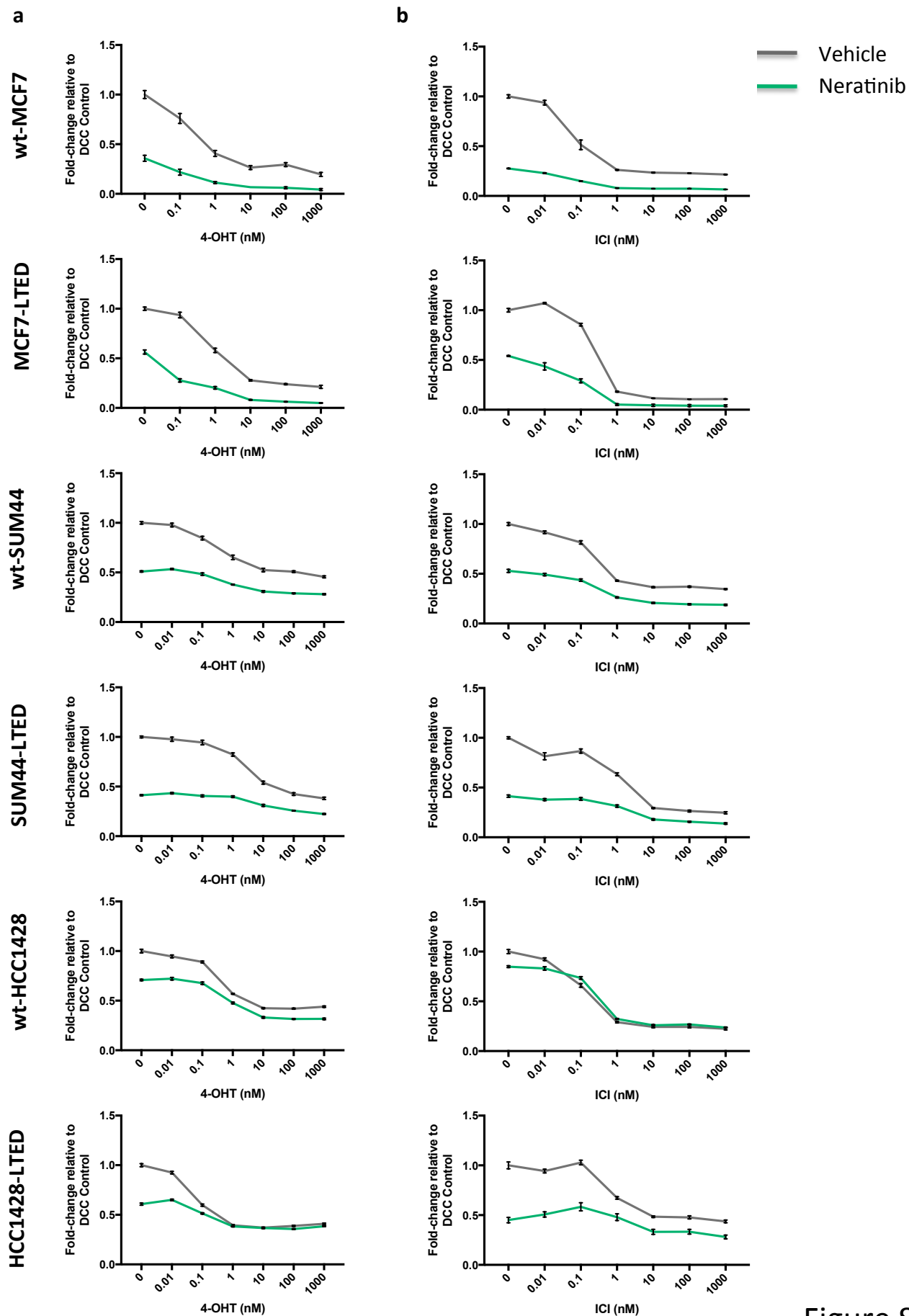

Figure S3

Supplement: Supplementary file 3 — Figure S3. Anti-proliferative effect of neratinib in combination with endocrine agents (a) 4-OHT and (b) ICI. Endocrine-resistant and -sensitive BC cell lines were treated with a combination of neratinib (500 nM in wt-MCF7 and MCF7-LTED; 700 nM in wt-SUM44, SUM44-LTED, and wt-HCC1428; 300 nM in HCC1428-LTED) and increasing concentrations of (a) 4-OHT or (b) ICI for 6 days with media change at day 3. Cell viability was analysed using a cell titer-glo assay. Data are expressed as fold-change relative to vehicle control. Error bars represent mean ± SEM. (PDF 195 kb) [file 13058_2018_983_MOESM3_ESM.pdf]

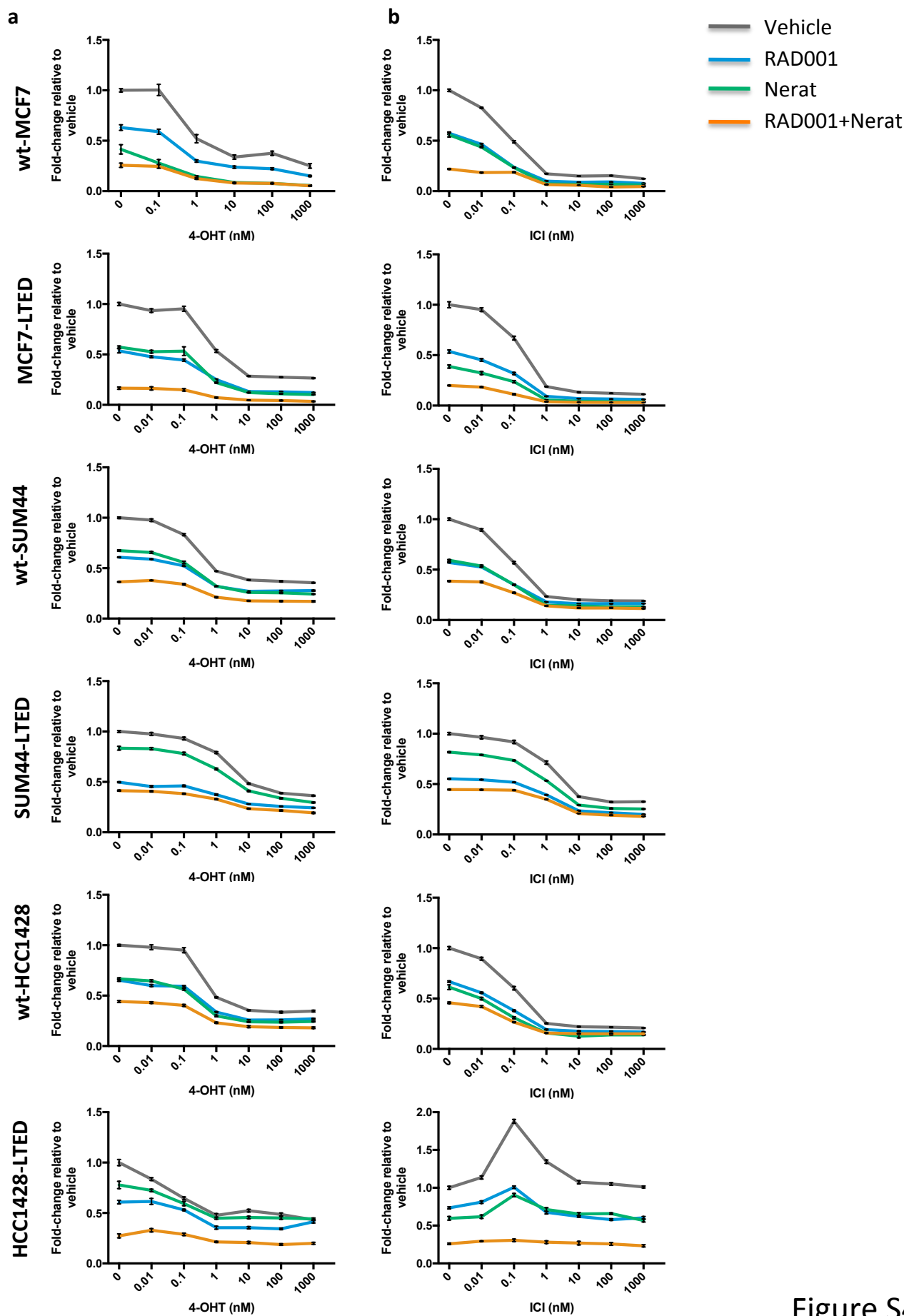

Figure S4

Supplement: Supplementary file 4 — Figure S4. Anti-proliferative effect combination of RAD001 and neratinib together with endocrine agents (a) 4-OHT and (b) ICI. Endocrine-resistant and -sensitive BC cell lines were treated with a combination of RAD001 and neratinib and increasing concentrations of (a) 4-OHT or (b) ICI for 6 days with media change at day 3. Cell viability was analysed using a cell titer-glo assay. Data are expressed as fold-change relative to vehicle control. Error bars represent mean ± SEM. wt-MCF7 (1.5 nM RAD001; 200 nM neratinib); MCF7-LTED (1.5 nM RAD001; 300 nM neratinib); wt-SUM44 (0.37 nM RAD001; 450 nM neratinib); SUM44-LTED (0.37 nM RAD001; 250 nM neratinib); wt-HCC1428 (1.5 nM RAD001; 500 nM neratinib); HCC1428-LTED (3 nM RAD001; 250 nM neratinib). (PDF 208 kb) [file 13058_2018_983_MOESM4_ESM.pdf]

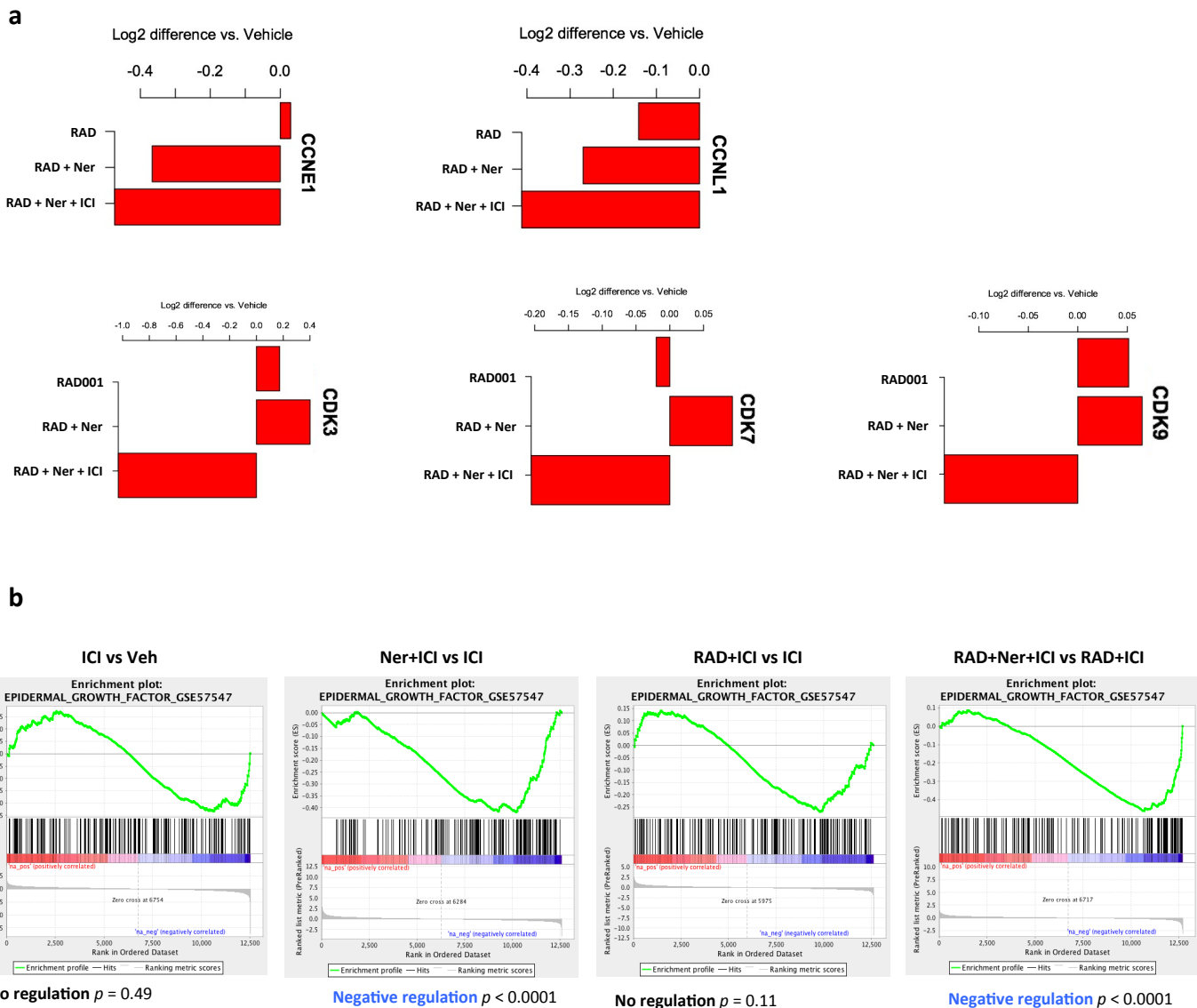

Figure S7

Supplement: Supplementary file 7 — Figure S7. Assessment of dynamic changes in expression of cell cycle regulatory genes. (a) Log2 differences in CCNE1, CCNL1, CDK3, CDK7, and CDK9 gene expression following treatment with RAD001, RAD001 + neratinib, and RAD001 + neratinib + fulvestrant (ICI), compared with vehicle. (b) GSEA enrichment plots for 198 genes known to be induced by sustained activation of ERK in response to EGF activity. Plots show the profile of the running Enrichment Score and positions of GeneSet Members on the Rank Ordered List for rank gene lists generated from the comparison of: ICI vs. vehicle; neratinib + ICI vs. ICI; RAD001 + ICI vs. ICI; and RAD001 + neratinib + ICI vs. RAD001 + ICI. (PDF 721 kb) [file 13058_2018_983_MOESM7_ESM.pdf]
