# Supplementary material for: Targeting tumour re-wiring by triple blockade of mTORC1, epidermal growth factor, and oestrogen receptor signalling pathways in endocrine-resistant breast cancer
Source: Breast Cancer Res. 2018 Jun 8;20:44. doi: 10.1186/s13058-018-0983-1 (PMC5992820; doi:10.1186/s13058-018-0983-1)
Supplement: Supplementary file 5 — Figure S5. Effect of RAD001, neratinib, or their combination with endocrine agents on ER-mediated transactivation and ER signalling. Cell lines were co-transfected with EREIItkLuc and pCH110, and treated for 24 h with the drug combinations indicated. IC50 values were used for RAD001 and neratinib together with standard concentrations of E2 (0.01 nM), 4-OHT (0.1 nM), and ICI (0.1 nM). Luciferase activity was normalized by β-galactosidase from triplicate wells and fold-changes expressed relative to the E2 control. Error bars represent mean ± SEM. *p < 0.05; **p < 0.01; ***p < 0.001. wt-MCF7 (2 nM RAD001; 500 nM neratinib); MCF7-LTED (4 nM RAD001; 650 nM neratinib); wt-SUM44 (3 nM RAD001; 700 nM neratinib); SUM44-LTED (3 nM RAD001; 700 nM neratinib); wt-HCC1428 (3 nM RAD001; 700 nM neratinib); HCC1428-LTED (10 nM RAD001; 300 nM neratinib). Western blot was used to assess changes in phosphorylation of the ER in response to RAD001, neratinib, or their combination together with endocrine agents. IC50 values were used for RAD001 and neratinib together with standard concentrations of E2 (0.01 nM), 4-OHT (10 nM), and ICI (1 nM) (with the exception of HCC1428-LTED where 10 nM was used). wt-MCF7 (2 nM RAD001; 500 nM neratinib); MCF7-LTED (4 nM RAD001; 750 nM neratinib); wt-SUM44 (3 nM RAD001; 700 nM neratinib); SUM44-LTED (3 nM RAD001; 700 nM neratinib); wt-HCC1428 (3 nM RAD001; 1000 nM neratinib); HCC1428-LTED (10 nM RAD001; 500 nM neratinib). (PDF 1240 kb) [file 13058_2018_983_MOESM5_ESM.pdf]

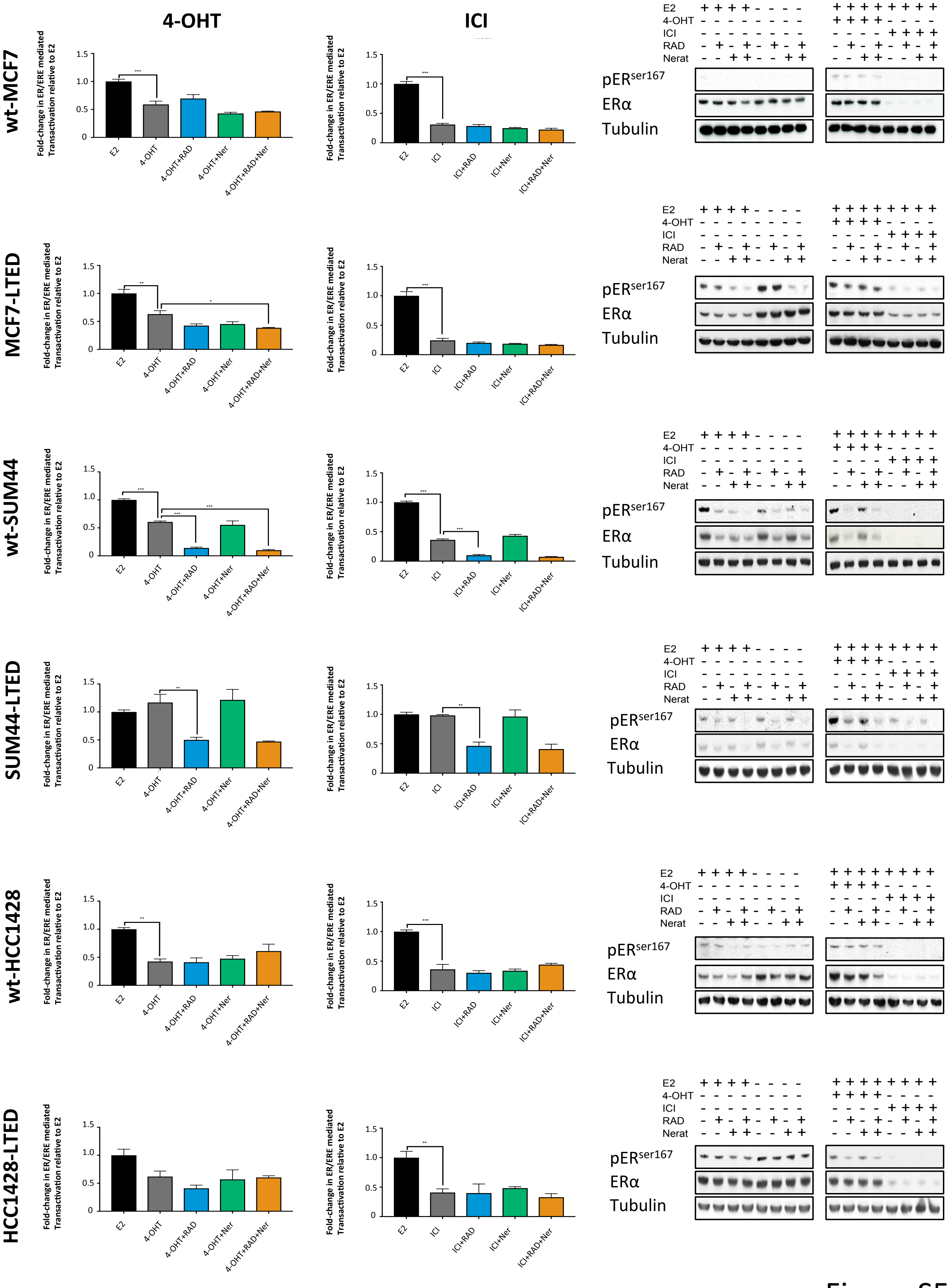
4-OHT
ICI
wt-MCF7
MCF7-LTED
wt-SUM44
SUM44-LTED
wt-HCC1428
HCC1428-LTED
Fold-change in ER/ERE mediated Transactivation relative to E2
E2
4-OHT
4-OHT+RAD
4-OHT+Ner
4-OHT+RAD+Ner
ICI
ICI+RAD
ICI+Ner
ICI+RAD+Ner
E2
4-OHT
ICI
RAD
Nerat
pER ser167
ERα
Tubulin

Figure S5
