# Supplementary material for: Targeting tumour re-wiring by triple blockade of mTORC1, epidermal growth factor, and oestrogen receptor signalling pathways in endocrine-resistant breast cancer
Source: Breast Cancer Res. 2018 Jun 8;20:44. doi: 10.1186/s13058-018-0983-1 (PMC5992820; doi:10.1186/s13058-018-0983-1)
Supplement: Supplementary file 6 — Figure S6. Assessment of dynamic changes in gene expression in response to RAD001, neratinib, or the combinations with fulvestrant. (a) MCF72a cells, which were previously engineered to express aromatase (CYP19) [33] were implanted into ovariectomised mice under androstenedione support. In this setting, MCF72a cells convert androstenedione in to oestrogen to drive proliferation. Once tumours developed, androstenedione was withdrawn. After a lag phase, tumour growth occurred synonymous with ligand independence. Assessment of the MCF72a-LTED showed continued expression of ER and proliferation in the absence of exogenous oestrogen providing a model of AI relapse. (b) Changes to gene expression (log2 difference drug – vehicle) as detected by RNA-seq for five drug combinations (neratinib, RAD001, ICI, neratinib + RAD001, and neratinib + RAD001 + ICI) were mapped to KEGG pathway graphs using Pathview (https://bioconductor.org/packages/release/bioc/html/pathview.html). Genes with a fold-change greater than 50% compared with vehicle in any condition were selected in order to expand the list of differentially expressed genes, allowing the identification of subtle changes in gene expression; for example, kinases or transcription factors that might have significant impact on downstream gene expression. A heatmap for each gene in shown. (c) Assessment of expression of E2F target genes in response to neratinib and RAD001. (PDF 1861 kb) [file 13058_2018_983_MOESM6_ESM.pdf]

a

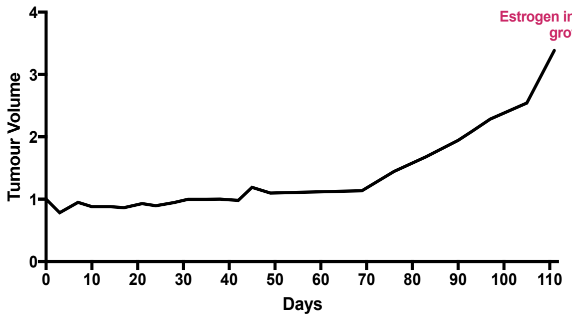

ER expression in MCF72a-LTED

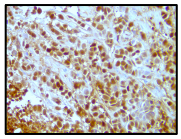

b

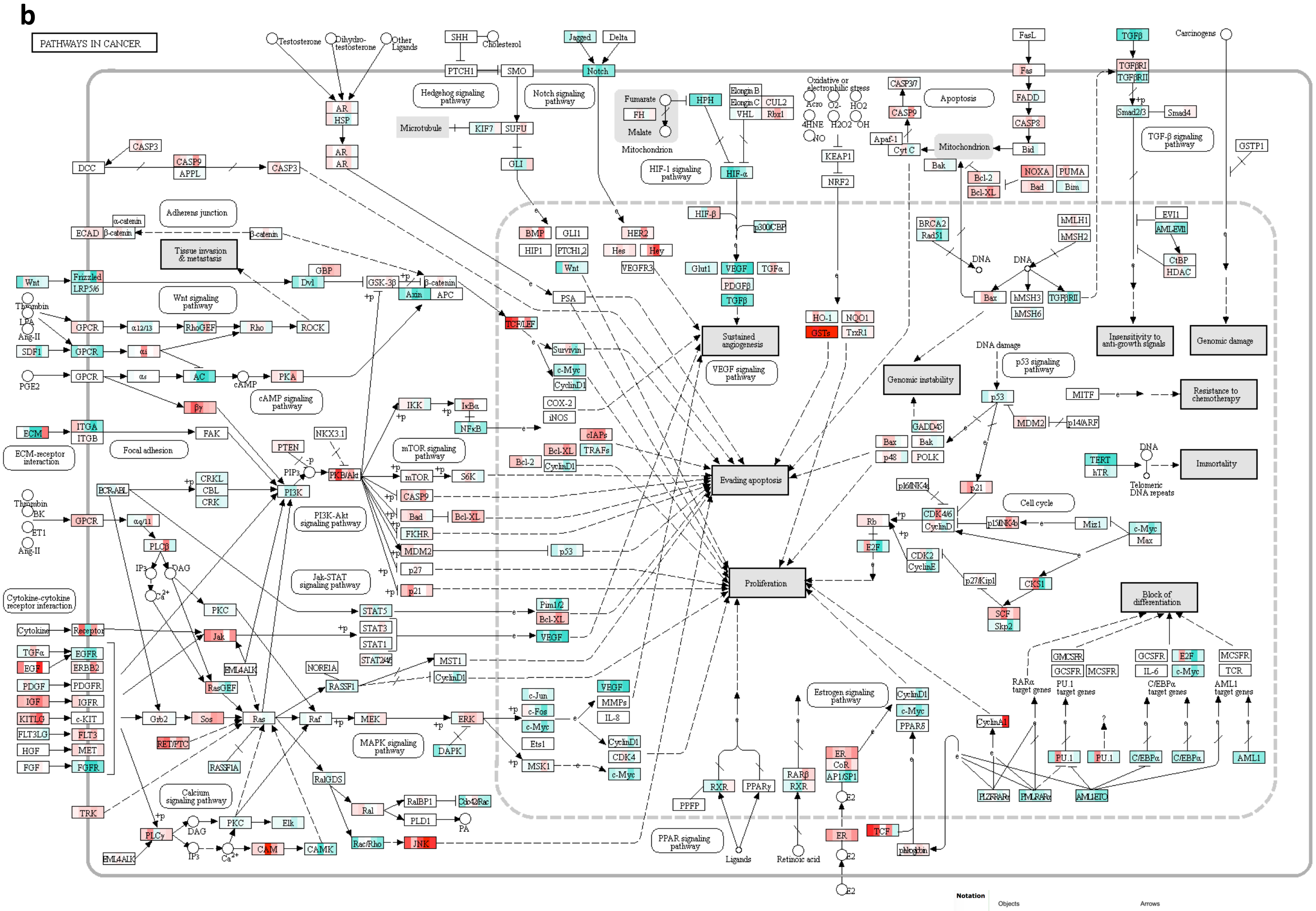

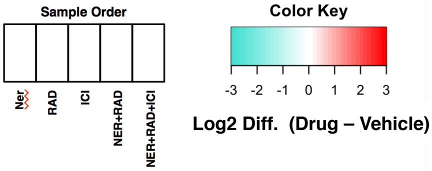

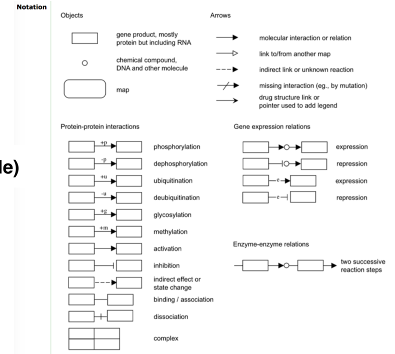

c

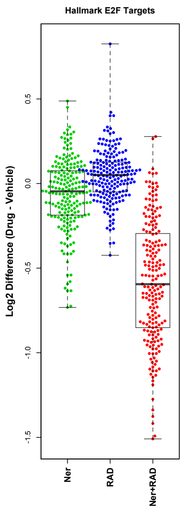

Figure S6
